# A qualitative research study on the illness perception of chronic pruritus in older Asian adults based on the Common‐Sense Model of self‐regulation

**DOI:** 10.1111/hex.13320

**Published:** 2021-07-26

**Authors:** Aminath Shiwaza Moosa, Natasha Sheng Yeng Leng, Chui Lien Kum, Ngiap Chuan Tan

**Affiliations:** ^1^ SingHealth Polyclinics Singapore Singapore; ^2^ SingHealth‐Duke NUS Family Medicine Academic Clinical Programme Singapore Singapore

**Keywords:** cause, chronic pruritus, common‐sense model, consequence, controllability, identity, illness perception, illness representation, older adults, time

## Abstract

**Background:**

Chronic pruritus (CP), itch lasting more than 6 weeks, is common in community‐dwelling older adults. Understanding their illness perception allows the attending physician to develop a personalised care plan to mitigate CP.

**Aim:**

This study explores the illness perception of CP among older Asian adults in an urban community.

**Design:**

Qualitative research was conducted, framed by the Common‐Sense Model of self‐regulation (CSM). Through in‐depth interviews (IDIs), qualitative data were gathered from Asian patients with CP, and then a thematic analysis was carried out. The emergent themes were grouped according to the five domains of CSM: ‘identity’, ‘cause’, ‘time’, ‘controllability’ and ‘consequence’.

**Setting and Patients:**

IDIs were conducted in a Singapore public primary care clinic before the data were saturated.

**Results:**

The CSM domains illustrate the illness perception of CP. CP was identified as a ‘problem’ rather than a disease and was often described in metaphor. Patients' perception of the cause was diverse due to the lack of provision of a clear explanation by their physicians. They opined that CP continued indefinitely. Without definite time to resolution, patients adapted their help‐ and health‐seeking behaviours to control it. The consequences included therapeutic experimentation, alternative therapy, self‐isolation, avoidance behaviours, emotional disturbance and dermatological complications.

**Conclusion and Patient Contribution:**

Patients provided information on their perception of CP, which aligned with the CSM. A multipronged approach is needed to deliver holistic and personalised care to patients with CP, providing clarity on its natural progression, to set their expectations on its timeline, treatment effectiveness and undertake appropriate behaviour modification to adapt to its chronicity.

## 1 BACKGROUND

Rapidly ageing populations are emerging in selected developed communities globally. Singapore is an example and is expected to have the ninth largest share of older persons in the general population by the year 2050.^1^ Older persons are defined as those aged 65 years and above.^2^ Ageing often leads to drying of the skin and changes in cutaneous nerve fibres. Older persons often have associated polypharmacy and multiple medical comorbidities. These constitute additional risk factors for itch or pruritus in these older persons.^3^

Pruritus is the most frequent cutaneous complaint in the geriatric population.^4^ It is a subjective symptom, which many older persons struggle to convey to others effectively.^5^ The causes are usually multifactorial and often misunderstood. Sommer et al.^6^ reported that 44.5% of patients had no specific single disease identified as the cause of their pruritus. Among these patients, mainly those aged above 65 years, multiple factors are likely to underpin their pruritus.^6^ This makes it more challenging to diagnose and treat the condition and leaves the patient unsatisfied. The Global Burden of Disease Study 2010^7^ reports a high prevalence of skin disease in persons older than 70 years of age, with pruritus being one of the top 50 interdisciplinary symptoms. The severity and chronicity of pruritus are significantly associated with poor sleep quality, more depressive symptoms, higher anxiety levels, more nonspecific somatic symptoms and impaired quality of life (QOL).^8^, ^9^, ^10^, ^11^, ^12^ Poor understanding of the cause and chronicity of CP may drive the patient to seek temporary symptomatic relief rather than regular preventive management of the condition. This is evident in other chronic diseases with periodical acute exacerbations such as asthma, with the disease pattern similar to that of CP.^13^

Chronic pruritus (CP) is defined as pruritus that persists for more than 6 weeks.^14^ The prevalence of CP increases with age.^15^, ^16^, ^17^, ^18^ Liao et al.^16^ showed that patients aged 65 years and older had a threefold increased risk of pruritus than those aged younger than 65 years. Ständer et al.^15^ found that the prevalence of CP increased with age from 12.3% (16–30 years) to 20.3% (61–70 years). The prevalence of pruritus in an inpatient geriatric patient population in Singapore is reported to be 48.5%, but its prevalence in the local community‐dwelling older adults has not been established.^9^

The impact of CP on daily life can be significant.^12^, ^19^, ^20^, ^21^ CP was shown to cause sleep disruption in 35.1% of older inpatients in a local tertiary hospital, while 30.9% reported difficulty concentrating on their daily activities.^9^ The impact of CP on the QOL is comparable to that of chronic pain, with the average patient willing to forfeit 13% of his or her life expectancy to live without the pruritus symptoms.^22^ The treatment of CP becomes critical in optimising the mental health and QOL of patients, but it remains challenging in clinical practice.^23^

Most local patients with CP are treated with sedating antihistamines, which increase their fall risks.^24^, ^25^, ^26^ Nonsedating antihistamines have a better risk and tolerability profile, but long‐term use is necessary to curb the symptoms, adding to the pill burden of the older patients.^27^ Prolonged symptomatic treatment leaves the patient unsatisfied as it fails to address the root of the condition. Symptom recurrence often leads to frequent doctor changes, adding to the healthcare cost.^5^ Frustrations with the treatment invariably affect their health‐seeking behaviour. Self‐treatment with over‐the‐counter medications, including the use of traditional medicated oils or powders to relieve their symptoms, is common among local patients with pruritus.^9^ Many of them rub or scratch the areas of pruritus, resulting in excoriations, or resort to frequent washing of the site to find transient relief.^20^ Many change their eating habits and adopt special diets to relieve their pruritus.^24^

These general measures, such as avoiding certain climates, topical irritants, diet and regular use of moisturisers, night creams in addition to relaxation therapy and educational programmes, has been shown to be effective in mitigating CP.^28^ Many studies demonstrate the effectiveness of regular emollients and moisturisers in managing pruritus with the need for symptomatic treatment during exacerbations.^23^, ^29^ A lack of understanding of the disease or the importance of treatment makes patients less likely to adhere to such a regime.^30^, ^31^ Dermatological conditions can be effectively controlled if patients understand the disease mechanism, trigger avoidance and follow proper skincare. Assessment of patients' perspectives to develop a baseline for education, evaluate motivation and ensure a complete understanding of the necessity for treatment has been reported to be important in previous studies of dermatological conditions.^32^, ^33^ Most conditions in dermatology have the advantage of patients directly observing the effects of treatment in terms of symptom relief or aggravation or status quo. Hence, the experience of following the treatment provides a feedback loop to reinforce illness and treatment perception. Thus, focusing the communication with the patient on their perceived illness and treatment and rectifying misbeliefs with evidence‐based information are relevant measures in managing CP. This approach may enable the patients to adapt their self‐care behaviour accordingly.^34^

Therefore, illness and treatment perception influence behavioural response, leading to harm if not managed prudently. Bathe et al.^5^ explored the illness perception of patients with CP in a German dermatological referral clinic. They reported that patients with CP feel misunderstood, as the latter struggle to describe their symptoms. These patients feel that their health professionals are insensitive to their illness burden. Invariably, some of them resort to consulting multiple doctors to seek clarification on their persistent symptoms. Others switch to complementary and alternative treatment modalities in a futile attempt to seek definitive therapy for CP.

Illness perception refers to patients' belief and expectations about an illness or somatic symptom. Illness perception refers to patients' belief and expectations about an illness or somatic symptom. The Common‐Sense Model of self‐regulation (CSM) has been developed to frame and facilitate the understanding of a person's illness perception via illness representation. In the model, individuals use three sources of information to make sense of and manage the problem: Information assimilated from the sociocultural environment; information from an authoritative figure such as a doctor or parent; and information obtained from current experiences with the illness. ‘Current experiences’ include existing perceptions, previous experiences with the illness and experiences associated with their coping measures. The combined information influences the individual to seek help, cope or adopt an illness management regime. The CSM model comprises five key domains: identity, timeline, cause, control/cure and consequences.^35^, ^36^, ^37^ It encapsulates the concept that the individual will use the three sources of information to identify the disease symptomatology, recognise the disease pattern over time and perceive the underlying causes, outcomes of the treatment and associated consequences.

In addition, cultural orientations and beliefs are important determinants of illness perception.^21^, ^36^, ^38^ Understanding the Asian cultural values, orientations and influences will provide insight into the local multi‐ethnic Asian patients' perception of their illness and reaction to their CP. Recognising the illness perception by patients enables their physicians to offer person‐centric approaches in both pharmacological and nonpharmacological management of pruritus and moderate their expectations of the clinical outcomes that may matter to patients.

This study aimed to explore the illness perception of CP in Asian patients and their behavioural and emotional responses framed by the CSM. The results can potentially translate to personalised care for these patients to receive explicit information and avoid harmful responses to CP.

## 2 METHODS

### 2.1 Research design

Qualitative descriptive research is deployed to explore patients' illness perception of pruritus, as it is subjective and likely multi‐dimensional. In‐depth interviews (IDIs) were conducted to collect the qualitative data pertaining to their experiences and the effect of CP on their activities of daily living.

### 2.2 Theoretical framework

CSM seems to be best suited to frame the study.^36^ This framework provides a comprehensive understanding of patients' perspectives of CP, covering their recognition of the illness (identity), aetiology perception (causes), belief of the illness duration (timeline), attributed outcomes (consequence) and observed treatment effects (controllability).

### 2.3 Study site

A public primary care clinic in Bukit Merah estate in southern Singapore was the study site. About 151,250 multi‐ethnic Asians live in this estate, which has one of the highest proportions of senior residents in Singapore. Approximately 1 in 5 residents is aged 65 years and older, totalling 33,310. About 38% of patients managed in this primary care clinic belong to this age group.^39^

### 2.4 Study population

The study population comprised multi‐ethnic Asian patients aged 65 years and older, who were invited to participate in the study if they could speak any local major languages. They self‐reported having experienced pruritus for longer than 6 weeks, regardless of aetiology and severity, with or without primary skin lesions or underlying disease. Those with physician‐diagnosed dementia, visual or auditory impairment and unable to provide written consent were excluded.

### 2.5 Recruitment procedure

Purposive sampling was carried out to recruit eligible patients from December 2019 till February 2021. Recruitment was suspended in February 2020 due to restrictions imposed during the COVID‐19 pandemic and resumed after approval from the institution in August 2020. Primary care physicians and healthcare workers at the study site were engaged to help identify suitable patients based on eligibility criteria. All potential patients provided verbal consent to share their contact details with the research team. The study team members subsequently contacted them to schedule face‐to‐face or phone interviews. Recruitment continued until data saturation was reached when no new themes emerged.^40^

### 2.6 Consent‐taking procedure

Informed written consent was obtained from patients before the interviews, which were conducted in a quiet room to ensure privacy. The patients read the study information sheet, consent form and topic guide before the interview. The documents were in English and were translated by a study team member for non‐English‐literate patients in a language and in layman terms so that they understood the nature of the study. A study team member addressed their queries before their consent endorsement.

### 2.7 Topic guide

A pilot topic guide was developed after literature review, discussion with the team members and keeping with the CSM framework. The pilot topic guide was reviewed and revised to create the final topic guide (Table 1). The latter included questions on the description and perception of the cause of pruritus, the expected duration and impact of pruritus on their daily activities, emotions and health‐seeking and self‐care behaviour. Questions were phrased to be easily understood by the patients and medical jargon was avoided.

**Table 1:** Topic guide

Questions	Framework domain
How would you describe your itch?	Identity
What do you think causes you to have itch?	Cause
How long do you think your itch will last?	Timeline
How does it affect your daily activities?	Consequence
What kind of emotions do you feel because of the itch?	
What issues have you encountered because of the itch?	
What issues have you encountered while treating the itch?	
Who and where do you get help to treat your itch?	Controllability
How do you treat your itch?	
What behaviour or habits have you changed to avoid your itch?	
What treatments and methods control your itch?	

### 2.8 Data collection and interview

The patients filled their demographic data before the face‐to‐face interviews or phone interviews, which was an additional approved option during the pandemic. Individual IDIs were conducted as personal and sensitive issues were expected to emerge, which were deemed inappropriate to share in group discussion.

The interviews, completed in 40–90 min, were audio‐recorded using a digital Dictaphone with a microphone attachment. The patients were remunerated with an SGD20 (USD15) grocery voucher for their time and contribution to the study. The audio recordings were translated or transcribed verbatim by professional transcribers. The transcriptions were next audited by an independent researcher for accuracy. Each patient was assigned a unique study identity number to ensure confidentiality and anonymity.

### 2.9 Coding and data analysis

Coding was carried out using an iterative approach. Three investigators coded the first three audited transcripts independently to generate an initial coding frame. Based on this initial coding frame, subsequent transcripts were coded by the first author using the software NVivo^©^ version 12 (QRS Pty Ltd). The qualitative data were regularly discussed among the investigators and the codes were reviewed, revised and refined based on the theoretical framework to identify emerging themes.

The demographic data collection forms, audio recordings, transcripts, coding framework and codes were maintained in secure archives to establish a clear audit trail.

### 2.10 Ethics considerations

The study was approved by the SingHealth Centralised Institutional Review Board (CIRB 2019‐2084) and was funded by the Family Medicine Academic Clinical Programme Seed Grant (PRACPR175808). The research was conducted according to International Conference on Harmonisation Guidelines for Good Clinical Practice.

## 3 FINDINGS

A total of 17 patients were approached and 13 consented to the study (response rate: 76.5%). The 13 IDIs consisted of 7 face‐to‐face and 6 telephone interviews. Those who declined participation cited time constraint, lack of interest or they felt they did not have valuable information to contribute. The patients' demographic characteristics are reported in Table 2. The results are presented according to the domains in the CSM. A summary and description of the themes are presented in Table 3.

**Table 2:** Demographic characteristics of the study population (*N* = 13)

Characteristics	Number	Percent
Age		
65–69	6	46.2%
70–74	4	30.8%
75+	3	23.1%
Mean age in years (72.4)		
Gender		
Male	8	61.5%
Female	5	38.5%
Ethnicity		
Chinese	10	76.9%
Malay/Indian/Eurasian	3	23.1%
Marital status		
Married	9	69.2%
Single	3	23.1%
Separated	1	7.7%
Highest education		
Primary	3	23.1%
Secondary	6	46.2%
A‐level/diploma	3	23.1%
University/posttertiary	1	7.7%
Current housing type		
HDB < 4 room flat	6	46.2%
HDB > 3 room flat	6	46.2%
Private condominium	1	7.7%
Income tax paid past year		0.0%
No	12	92.3%
Yes	1	7.7%
MediFund^a^ assistance		
No	12	92.3%
Yes	1	7.7%

Abbreviation: HDB, Housing and Development Board (Singapore's public housing which is managed by the Housing and Development Board).

^a^ MediFund assistance is an endowment fund for lower‐income Singaporeans who are unable to pay for their medical expenses.

**Table 3:** Themes and subthemes

Themes	Description of themes	Subthemes
IDENTITY	Identification and description of pruritus	Problem described in the metaphor
CAUSE	Perceived causes and triggers of chronic pruritus	Diffused, unclear cause due to lack of explanation
TIMELINE	Belief about the expected duration of chronic pruritus	Indefinite course and expectation
CONSEQUENCE	Physical and emotional impact of chronic pruritus on daily living	Isolation and avoidance behaviour
		Sleep disruption
		Dermatological complications
		Emotional disturbance
CONTROLLABILITY	Help‐ and health‐seeking behaviour to manage the chronic pruritus	Scratch response
		Help‐seeking behaviour
		Health‐seeking behaviour with a western doctor
		Alternative therapy with herbs, oils and powder

### 3.1 Identity

#### 3.1.1 Problem described in metaphor

The patients struggled to identify and describe their symptoms, generally as an unpleasant sensation. They tended to use metaphor in their narration of their experience:

‘Alamak (local colloquial exclamation)! The fungus so great, like worms crawling!’ P4

‘If it gets worse, it gets reddish. It's like poking, poking pain like that, you know!’ P5

The patients referred to pruritus as a major problem, which continued to puzzle and trouble them.

‘It (pruritus) is a big problem…’ P9

‘So, I still don't know why, … that is the problem that I'm living with now. I don't know WHAT is the problem. So, if I don't know what is the problem, how can I find a cure for it?’ P3

### 3.2 Cause

#### 3.2.1 Diffused, unclear cause due to lack of explanation

None of the patients had identified the cause of their pruritus.

‘…My wild imagination. You have no knowledge exactly what causes it lah (colloquial), whether it's dry skin or old age or what!’ P4

The patients highlighted the lack of explanation by their attending physician, resulting in their own perception of its aetiology. Some of them attributed it to external factors like environmental temperature and cleanliness, water quality, skin products, type and colour of clothing.

‘…both (doctors) actually didn't explain to me how this thing come about and what I shouldn't take or what I shouldn't do, you know? But I wish to know that, if possible’. P2

‘Supermarket aircon lah! you go to the seafood area, the aircon exceptionally cold, right?… very fast feel itchy because maybe the air not clean or what? I feel exceptionally itchy’. P11

‘You don't wear black. Black doesn't suit… because black you go outside, hot right? It (absorbs) the heat and you can feel the itch’. P7

Other patients attributed CP to ageing, medical conditions, medication or consumption of certain foods.

‘Any other medical underlying problem, maybe like diabetes, then you can have … this itch. Yah’. P5

‘People advise me, don't take salt or take less salt… maybe you eat too much salt, you get this lah! because the salt goes into your body … duck meat, laksa (a local spicy noodle), all these MUST NOT take’. P12

‘Most of my friends were telling me once a woman, after menopause, they got a lot of problem… like dry skin. So once you have dry skin, you have itchiness then I believe it's due (to) less…Estrogen … hormone. People when they grow older, the skin becomes so dry, and then there is itchiness’. P7

### 3.3 Timeline

#### 3.3.1 Indefinite course and expectation

While most of the patients expected the CP to persist indefinitely, some remained hopeful of a cure.

‘I don't know … I think until I die lah!’ P13, when asked about the duration of the CP.

‘I only hope … if (I apply) my cream every time, and … with the tablet… if it really can cure me totally, I'm very, very happy’. P7

### 3.4 Consequence

#### 3.4.1 Isolation and avoidance behaviour

CP affected the patients in their daily living, including work, exercise and attire. To avoid worsening of CP, the patients isolated and confined themselves to indoor activities, avoided extremes of temperature, refrained from scratching in public, hid their wound and became selective in their food, clothing, jewellery and cosmetics.

‘Each time I go out, I make sure I have a good bath using a lot of (selected brand of emollient) first… I must prepare myself, give myself more time to prepare before I go out. I dare not scratch in public lah! My goodness!’ P4

‘So physically, I try to limit my activities. I used to go for walks, when I perspire, it gets worse. When I do housework, it gets worse. Yah! So maybe (it's) the heat. But I don't know! When in the cold country, the rash doesn't clear (up). Perfume also, I'm allergic! Also the jewellery, when I wear necklaces, especially those metal ones, yah, cannot… so my jewellery is usually plastic or wood. Even for shampoo, I also don't use the “anti (hair)fall” … all those strong ones, I don't use. When I go travelling, those disposable panties, not cotton ones, cannot!’ P5

‘I just stay at home and look after my leg… I just take care of it. I (am) scared I (would) scratch … that's why I don't want to go out… wound so ugly!’. P9

#### 3.4.2 Sleep disruption

Sleep was disturbed significantly due to the CP. Patients reported difficulty in both falling asleep and frequent sleep interruptions because of the pruritus.

‘Sometimes, at night, I can't sleep at all. Every two hours or one hour, you got to wake up’. P1

‘I (am) scared … I worry… that's why I cannot sleep at night. (In the) morning (when I) wake up … Alamak! All become wound already’. P9

#### 3.4.3 Dermatological complications

Patients reported skin complications secondary to scratching, including excoriations, bleeding, infection, skin thickening and scarring. Complications also arose from their self‐treatment.

‘The more you scratch, the skin gets thicker. It's very itchy … after you scratch, scratch until the skin (is) broken, it become very warm’. P12

‘(a brand of antiseptic solution) … I didn't feel the burn but … my skin started to turn blackishó’. P3

#### 3.4.4 Emotional disturbance

Depression, frustrations, embarrassment, anger, guilt, fear and sadness were some of the emotions alluded by the patients.

‘yes, because itch is no joke, you know [laughs]. Yah, it's one thing you cannot control and it causes you, aiyah (local colloquial term for frustration), a lot of embarrassment and problem’. P4

‘Just anxious that it keeps recurring’. P13

### 3.5 Controllability

#### 3.5.1 Scratch response

Scratch was the usual response to the itch, ranging from pinching, poking, scratching to rubbing with fingers or against furniture or other surfaces. Clothes, paper clips, scratch‐sticks or chopsticks were used to scratch the out of reach sites of the body. Though the scratch response was initially regarded as gratifying, the patients felt that it ‘trapped’ them into a vicious ‘scratch–itch’ cycle.

‘Once you scratch it, you fall into the trap already’. P11

#### 3.5.2 Help‐seeking behaviour

Many patients sought assistance from family and friends, pharmacists, doctors and alternate medicine practitioners. They searched for information from various media such as radio, television, newspaper and the internet. One patient mentioned sharing the experience with friends in managing the pruritus.

‘I have friends who have rash problem also. They tell me, recommend me this, recommend me that, and we try to exchange ideas’. P5

#### 3.5.3 Health‐seeking behaviour with a western doctor

The patients self‐medicated and avoided seeking medical attention when some of them perceived the condition as not severe to warrant a physician consult:

‘I just self‐medicate …I buy the cream, I apply it and then if it comes back again, (I) then just apply it, just leave it like that. I don't want to make myself so troublesome to do all this (by) seeing doctor, so many doctors’. P7

Some patients were reluctant to seek medical help due to the experience of an inconsistent treatment regimen. Failed symptom resolution prompted some patients to consult various doctors in different care settings. However, the outcomes remained disappointing and they did not attend further follow‐up.

‘If I go this month, they (doctors) give me (one) type of cream, then when I go another month, another doctor gives me another type of cream. So, it will be more confusing, you know’. P1

‘Many, many years … the doctor keeps saying it's dry skin. Then, I went to this near neighbourhood clinic, they (give) injection. So, after one, two times, I find that it's not changed. The doctor never helps me to solve problem… Injection no good. You inject something into my body, then next time I have to depend on this injection a lot, no good, right? So, I stopped going there (GP clinic). Then, I ever go to (tertiary dermatological referral center) to see doctor. Skin doctor gave me that type of pills, also don't help, so I stopped going, stop follow‐up’. P11

Few patients reported a beneficial effect of moisturising cream and continued using it regularly, although it did not cure the pruritus. They also reported unpleasant side effects and high cost of certain brands of emollients.

‘I was introduced to this (selected brand of emollient)Yah, that saved my life! It helped. I've been faithfully using quite a few bottles. And then I experimented and tried not to use it at night. For the first two or three nights, for not using, eh, it worked. Wow, no itch, nothing. But then, it came back (on) one of the nights, so I started using (it) again. So now I take (it on) alternate nights and it helps’. P4

‘They (doctors) ask me to apply moisturiser ALL OVER the body, but. the cream, you don't feel comfortable, you know, very sticky!’ P1.

‘After the injection, I feel very groggy…’. P5.

‘I feel that it's (selected brand of emollient) very expensive, so I just use the normal moisturiser… (from) polyclinic’. P10

#### 3.5.4 Alternative therapy with herbs, oils and powder

Some patients used alternative treatments such as oral herbs, over‐the‐counter topical cream, powder and massage oil to relieve their pruritus. Nonetheless, some patients were hesitant to use alternative treatments.

‘I went for body massage. It's better, you know. The patch is not so big. After applying the medicine, the itchiness patch will be more spread out…it's better. Less itch’. P1

‘He (traditional physician) gives me some Chinese herbs. After I take the Chinese herbs, I feel okay, but (itch) comes back again’. P10

‘I use the … “snake powder” [referring to prickly heat powder”]. cooling. It's VERY good. Especially after you bathe, the body (is) a little bit wet, you apply it, dissolve… it's very good. It will take away the itchiness’. P12

‘Ah, no, no, I haven't tried it (Traditional Chinese Medicine) yet. I actually wanted to try, but I didn't. I don't know why’. P2

## 4 DISCUSSION

The CSM appears to adequately depict patients' illness perception of their CP. Their understanding of CP, the resultant coping strategies and subsequent outcomes were covered in this model. Patients' cognition of the identity, perception of cause and chronicity of CP influenced their behaviour of controlling the ‘problem’ and various consequences.

This study sheds light on weaknesses in patient–physician communications, similar to that of findings from an exploratory study on adult patients with CP carried out by Bathe et el.^5^ Patients' accounts suggest a lack of clear and consistent information provided by the physician, resulting in patients struggling to describe the illness and understand its aetiology, expectations, management and effective means of coping with CP.

Patients tend to speculate on the exact type of CP they have. They defined pruritus as a vague and aversive sensation. Some of them labelled it as a ‘problem’, while others used metaphors to describe the CP. Their variable descriptions, including the metaphors, may confuse, mislead and potentially result in misdiagnosis by the attending physician. Patients themselves struggle in understanding the puzzling sensations. Using a metaphor to describe the symptom in the form of a third‐person narrative and seeking concordance from the patient may be an approach to engage the patient. For example, a physician may pose a question like: ‘Some patients with itch describe the sensation to be like “worms”, do you feel the same way?’. Putting the patient on common ground with other similarly affected persons may put them at ease to continue discussing their experience, treatment and coping plan.

Patients appear to connect the cause, symptoms and the underlying pathophysiology of the disorder as stipulated in the CSM. A common understanding among the patients is that ageing leads to dry skin, underpinned by lack of moisture. Those patients with a linked model were clear and successful in controlling the pruritus by regular moisturising of the dry skin. When there is uncertainty on the cause, due to patients' inability to understand the association mentioned above or lack of provision of information by physicians, patients tend to try various treatment modalities and often become dissatisfied with the outcomes. A qualitative study on patients with scabies similarly revealed uncertainty about their illness and diagnosis, poor understanding of their illness‐related events and insecurity.^41^ In contrast, sharing information about illness is known to improve the doctor–patient relationship and patient satisfaction.^42^ According to Mishel's Theory of Uncertainty in Illness, healthcare professionals can reduce the uncertainty by providing information on the causes and consequences of symptoms and increase a patient's clarity and knowledge of the illness.^43^

The recurring symptoms led patients to believe that CP has an indefinite and undetermined chronic course. Symptomatic treatment with antihistamines provides a transient improvement in pruritus, while regular use of emollients helps to control the pruritus. Clarity on the chronicity of the condition is crucial to dispel the misperception of and attribution by patients to an acute illness model.^13^ This will facilitate their adherence to preventive measures such as daily emollient application and avoid futile searches for alternative treatment modalities. Thus, instead of inconsistent and confusing regimes, the physician should adopt a more pragmatic approach to provide a clear and actionable chronic disease care plan to patients to manage their CP. Akin to the asthma action plan,^44^ the CP care plan should cover daily use of ‘controller’ topical application of moisturiser and promptly use ‘rescuer’ therapy with topical corticosteroid and adjust their antihistamine dose during acute itch exacerbations.

The controllability domain in CSM provides insight into patients' health behaviour in relation to their illness perception and treatment effect. Patients tend to adhere to the prescribed CP care plan when they recognise the benefit. In addition, educational interventions have been proven to improve treatment adherence. Hence, physicians need to provide consistent factual information to guide patients in their therapy.^34^

The controllability also provides a context to frame the coping behaviour. The disappointing clinical encounters result in patients attempting to self‐manage their condition using various coping mechanisms. Symptom control results in problem‐focused coping behaviours such as taking a shower and using a moisturiser before outdoor activities. In contrast, some patients resort to avoidance coping strategies, including the tendency to confine oneself to indoor activities when one fails to control their CP. Such behaviours lead to consequences that impact their daily lives, social functioning and emotional well‐being. This negative impact on patents' well‐being is observed in pruritus associated with dermatological and systemic conditions.^45^ Implementation of a nursing programme targeting patients with CP (‘Coping with itch’) has demonstrated effectiveness in reducing helpless behaviour and psychosocial morbidity.^46^, ^47^ By incorporating psychological and behavioural therapy, such a structured programme enables patients to adopt appropriate coping behaviour and avoid harmful response to CP.

### 4.1 Clinical relevance and impact

This study draws attention to the fact that physicians should clarify the type and cause of CP with patients, provide information on its chronicity and develop an action plan to control their symptoms and avoid adverse consequences. The uncertainty experienced by patients can be reduced by providing clear information on the cause, course, control and treatment expectation of CP. Targeted counselling, education and psychological support are integral in their care plan to increase their knowledge of CP, and strengthen and sustain appropriate coping behaviours.

### 4.2 Strengths and limitations

This study illustrates the illness perception of Asian patients of CP as a matter of common sense in self‐regulation. Use of the CSM represents a strength of this study. The domains of identity, cause, timeline, controllability and consequences deepen our understanding of the patient's awareness, experience and coping behaviour towards CP. The CSM will also serve as a model to develop a programme to train clinicians and other healthcare professionals to manage illness perception and address the specific issues in each domain.

There are limitations in contextualising the findings to a specific theoretical framework as some of the findings that may relate to illness perception may not be captured adequately. Considering the breadth of the findings based on CSM, these findings are sufficient to induct the physicians on the illness perceptions via illness representations of CP and their application in clinical practice to address the issues in most patients with CP. Despite purposive sampling, the study population mainly included Chinese patients. Variations in the perception of CP by other ethnic groups may be under‐reported. Most of the patients were highly educated and understood the rationale for the study. The coping and avoidance behaviours of those with CP who were less educated might differ. Nonetheless, the results cannot be generalised to the local Asian population, but provide adequate data to design a subsequent survey to assess the magnitude and morbidity of CP.

## 5 CONCLUSION

This study yielded in‐depth information on the domains of illness perception of Asian patients with CP and how patient–physician encounters drive the perception of this illness. They identified CP as a chronic, aversive problem with an unclear cause and infinite course, which impacted their daily lives and emotional well‐being. These perceptions were perpetuated due to the lack of information provided by physicians, resulting in problem‐focused or maladaptive behaviours. These conclusions emphasise the need for physicians and healthcare providers to rectify the illness perception, and educate and guide patients to adapt and adopt appropriate behaviours to mitigate adverse consequences.

## CONFLICT OF INTERESTS

The authors declare that there are no conflict of interests.

## AUTHOR CONTRIBUTIONS

The research team included three family physicians, one nurse and one medical student trained in qualitative research. The family physicians manage and counsel patients in polyclinics, including those with chronic pruritus. Ngiap Chuan Tan is a trainer and experienced researcher in both qualitative and quantitative research. Aminath Shiwaza Moosa facilitated all the interviews and took field notes, while the other investigators assisted in obtaining consent and in demographic data collection. Aminath Shiwaza Moosa, Natasha Sheng Yeng Leng and Chui Lien Kum coded the qualitative data. Aminath Shiwaza Moosa and Ngiap Chuan Tan analysed the data and identified the themes according to the Common‐Sense Model of self‐regulation. Aminath Shiwaza Moosa drafted the manuscript. Ngiap Chuan Tan and Aminath Shiwaza Moosa revised the draft several times before it was finalised. All investigators reviewed and approved the final manuscript before its journal submission.

## Data Availability

The data that support the findings of this study are available from the corresponding author upon reasonable request.
